# Lung Ultrasound Findings in Patients with COVID-19

**DOI:** 10.1007/s42399-020-00553-0

**Published:** 2020-10-01

**Authors:** Daniel T. Marggrander, Frauke Borgans, Volkmar Jacobi, Holger Neb, Timo Wolf

**Affiliations:** 1 Faculty of Medicine, J. W. Goethe University, Theodor-Stern-Kai 7 (H33 C), 60590 Frankfurt am Main, Germany; 2 Department for Infectious Diseases, Centre of Internal Medicine II, University Hospital Frankfurt, Theodor-Stern-Kai 7 (H68-2), 60590 Frankfurt am Main, Germany; 3 Institute of Diagnostic and Interventional Radiology, University Hospital Frankfurt, Theodor-Stern-Kai 7 (H23 C), 60590 Frankfurt am Main, Germany; 4 Department of Anesthesiology, Intensive Care and Pain Medicine, University Hospital Frankfurt, Theodor-Stern-Kai 7 (H23 C), 60590 Frankfurt am Main, Germany

**Keywords:** COVID-19, Lung ultrasound, SARS-CoV-2, Interstitial pneumonia

## Abstract

The current SARS-CoV-2 outbreak leads to a growing need of point-of-care thoracic imaging that is compatible with isolation settings and infection prevention precautions. We retrospectively reviewed 17 COVID-19 patients who received point-of-care lung ultrasound imaging in our isolation unit. Lung ultrasound was able to detect interstitial lung disease effectively; severe cases showed bilaterally distributed B-Lines with or without consolidations; one case showed bilateral pleural plaques. Corresponding to CT scans, interstitial involvement is accurately depicted as B-Lines on lung ultrasound. Lung ultrasound might be suitable for detecting interstitial involvement in a bedside setting under high security isolation precautions.

## Introduction

SARS-CoV-2 is a coronavirus that emerged in December 2019 in Wuhan city, Province Hubei, China [1] that causes COVID-19, a viral disease that causes influenza-like illness, which can progress into pneumonia [2]. The evaluation of the extent of pulmonary involvement using radiographic imaging is problematic due to infection prevention and control (IPC) measures that must be taken and the sheer number of cases that need evaluation. Critically ill patients, especially those treated with mechanical ventilation and extracorporal circulation devices, are particularly difficult to transport in order to obtain computed tomography (CT-) scans, and bedside chest x-ray (CXR) remains an unreliably inaccurate and unsatisfactory imaging modality [3, 4]. Point-of-care ultrasound imaging however is available at the patients’ bedside and accelerates diagnostics in respiratory distress in comparison to radiographic imaging [5]. Reproducible imaging artifacts in lung ultrasound correspond to underlying conditions of pulmonary tissue. The healthy lung surface yields the sonographic image of pleural gliding as well so-called A-Lines, horizontal reverberations of the pleural line. Thickened interlobular septa, for instance in edema, fibrosis, or infectious disease [6], cause B-Lines on LUS [7]. Those are comet tail-artifacts, vertical lines arising from the pleural line, extending to the bottom of the screen and moving with respiration. Three or more B-Lines per intercostal space are considered pathological, less than three (indicated by a lowercase “b” [8]) may occur in healthy individuals without corresponding to pathology. A *tissue-like* pattern is observed on ultrasound in the absence of air, with irregular borders adjacent to aerated lung, the so-called *shred sign*, occurring for instance in pneumonic consolidation, atelectasis, pulmonary embolism (PE) or solid tumors. Dynamic detection of air within the bronchi or perfusion detected by color Doppler may help differentiate these signs further [4]. In intensive care, lung ultrasound (LUS) can be used as bedside imaging without disadvantageous effects on patient care while reducing chest radiography and CT-scans [9–11]. LUS is useful in detecting interstitial pulmonary pathology [4] including interstitial pneumonia, which is the most common finding in patients with SARS-CoV-2 infection and pulmonary involvement [12]. A recently published letter describes the use of LUS and the typical ultrasonographic appearance in COVID-19 patients in Xiangya, Hunan, and in Beijing [13]. Due to the recent emergence of the disease and ongoing disease burden worldwide, we decided to share our experience with point-of-care bedside LUS in patients with COVID-19 in our department and provide a comparison of common LUS imaging and their corresponding findings on chest CT, as well as unusual LUS findings in COVID-19.

## Methods

We retrospectively reviewed LUS examinations that we performed in our isolation unit in patients who tested positive in polymerase chain reaction (PCR) of oral and nasal swabs for SARS-CoV-2. All patients received thorough scanning of their anterior, lateral, and posterior chest wall using either an *APLIO 300 TUS-A300* ultrasound system (Toshiba, Tokyo) with a 3.5 MHz convex probe or a *NEMIO SSA-550a* ultrasound system (Toshiba, Tokyo) with a 3.75 MHz convex probe. We assessed at least 4 intercostal spaces in both the anterior and lateral chest wall. In all but two patients, who were in critical condition and could not sit upright, we assessed at least 6 intercostal spaces in the posterior chest wall. In the supine patient, the probe was placed perpendicularly to the skin in each intercostal space, starting at the parasternal line, and moved laterally to obtain views of large portions of the lung surface. Posterior lung scans in the sitting patient started at the paravertebral line, moving laterally. All scans were strictly performed in longitudinal orientation (with the index of the probe facing towards the patients’ head). We assessed the appearance of A-Lines, B-Lines, consolidations, and pleural abnormalities. Either TW (attending physician) or DM (doctoral candidate) performed LUS and discussed the obtained images with the other ultrasound examiner to reach a consensus. Both TW and DM have had 3 years of experience in LUS, focusing on LUS in pulmonary infections.

If available, we reviewed corresponding conventional imaging that was performed in these patients for comparison. CXR was performed in posterior-anterior projection and lateral projection in stable patients, or in anterior-posterior projection as bedside radiograph in critically ill patients. Chest CT was performed without contrast agent for assessment of pulmonary SARS-CoV-2 involvement, or with contrast agent in critically ill patients where PE posed a probable differential diagnosis. Conventional imaging was evaluated by residents in our department of radiology and by their supervising attending physicians, who reviewed and discussed the images together with physicians from the infectious disease department.

We also reviewed the necessity of oxygen supplementation, mechanical ventilation, and auscultation findings in these patients.

## Results

We performed LUS in 17 COVID-19 patients (see Table 1) between the 6th of February and 1st of April 2020. The patients’ age ranged from 30 to 68 years (median 51 years). CXR was performed in 13 patients; chest CT was performed in eight patients. In 2 patients, we did not perform any radiological imaging.

**Table 1:** Characteristics of 17 patients: Results from LUS and radiographic imaging as well as clinical condition on admission (supplementary O_2_ flow, SpO_2_, auscultatory crackles, and necessity for mechanical ventilation [MV]). Consolidations on LUS are considered, “small”, when they do not extend over more than one ICS

Age (years)	Gender	SpO_2_	Crackles on auscultation	Supplementary O_2_ [min^−1^]	MV	Radiographic imaging	LUS findings
44	F	95%	None	None	No	CXR: Inconspicuous	A-Lines, small subpleural consolidation
58	M	98%	None	None	No	CXR: Inconspicuous	A-Lines, bilateral pleural plaques (Fig. 3)
52	F	96%	None	None	No	CXR: inconspicuous CT: GGO	A-Lines, small pleural effusion (4 mm)
59	M	94%	None	1 l	No	CXR inconspicuous CT: inconspicuous	A-Lines
31	M	96%	None	None	No	CXR: inconspicuous	A-Lines
32	M	96%	None	None	No	CXR: inconspicuous	A-Lines, small subpleural consolidation
43	M	97%	None	None		CXR: inconspicuous CT: GGO	A-Lines
30	M	94%	Present	10 l	Yes (ARDS)	CXR: inconspicuous CT: GGO, Severe bilateral consolidation	Predominantly B-Lines, large consolidation
49	M	94%	None	4 l	No	CT: GGO, consolidation	Predominantly B-Lines, small subpleural consolidation
68	F	95%	None	None	No	CXR: inconspicuous	Predominantly A-Lines, posterior B-Lines and large consolidation
30	M	*N.A*	None	None	No	None	A-Lines, small subpleural consolidation
54	F	*N.A*	None	None	No	None	Predominantly A-Lines, posterior B-Lines and large consolidation
59	F	89%	Present	6 l	No	CXR: opacities CT: GGO, consolidation	Predominantly B-Lines
51	M	97%	None	None	No	CT: Subtle GGO	Predominantly B-Lines, small pleural effusion (2 mm)
68	F	93%	None	1 l	No	CXR: opacities	Predominantly A-Lines, posterior B-Lines and large consolidation
68	M	98%	None	3 l	No	CXR: opacities	Predominantly B-Lines
37	M	99%	None	None	No	CXR: opacities CT: GGO, consolidation	Predominantly B-Lines, small pleural effusion (6 mm)

### LUS results

Of the 17 patients, only 3 showed no pulmonary abnormalities at all, meaning that we saw A-Lines in all assessed intercostal spaces (ICS), no more than 2 b-Lines in any intercostal space, no irregular pleural line, and no consolidation.

In 3 patients, we found small consolidations (no more than one ICS in longitudinal extent) with or without adjacent comet tail artifacts, but no ICS with 3 or more B-Lines.

One patient had a small pleural effusion with a sagittal extent (between lateral chest wall and diaphragm) of 4 mm unilaterally, but no ICS with 3 or more B-Lines and no consolidation.

**Fig. 1:**
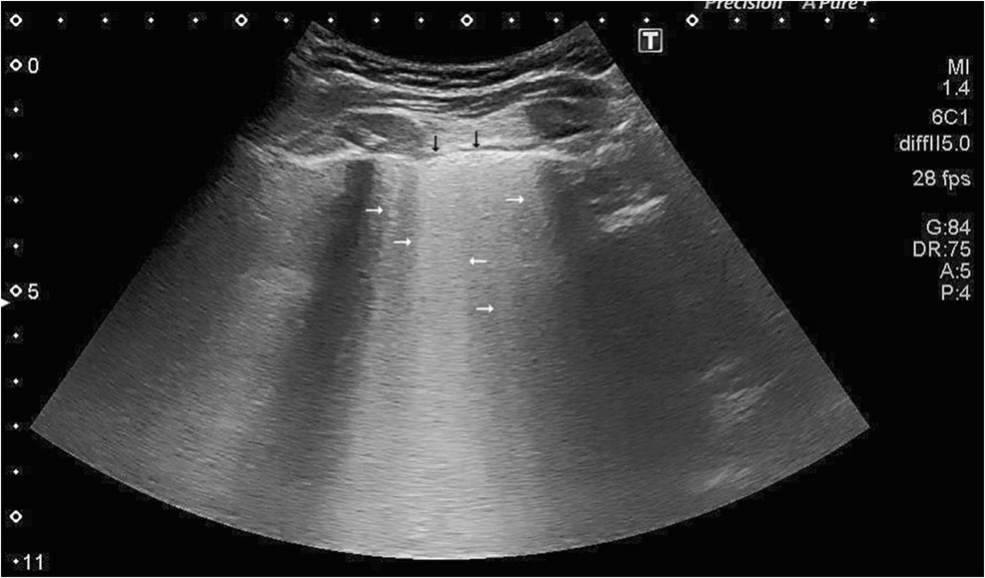
Coalescent B-Lines in a COVID-19 patient: Hyperechoic artifacts (horizontal arrows) arising from the pleural line (black arrows) and extending vertically (in regard to the screen) to the bottom of the image, moving with the cycle of respiration. Any *horizontal* artifacts below the pleura that are usually seen in the healthy lung and represent reverberations of the pleural line (A-Lines) are obliterated by B-Lines, and are absent here

**Fig. 2:**
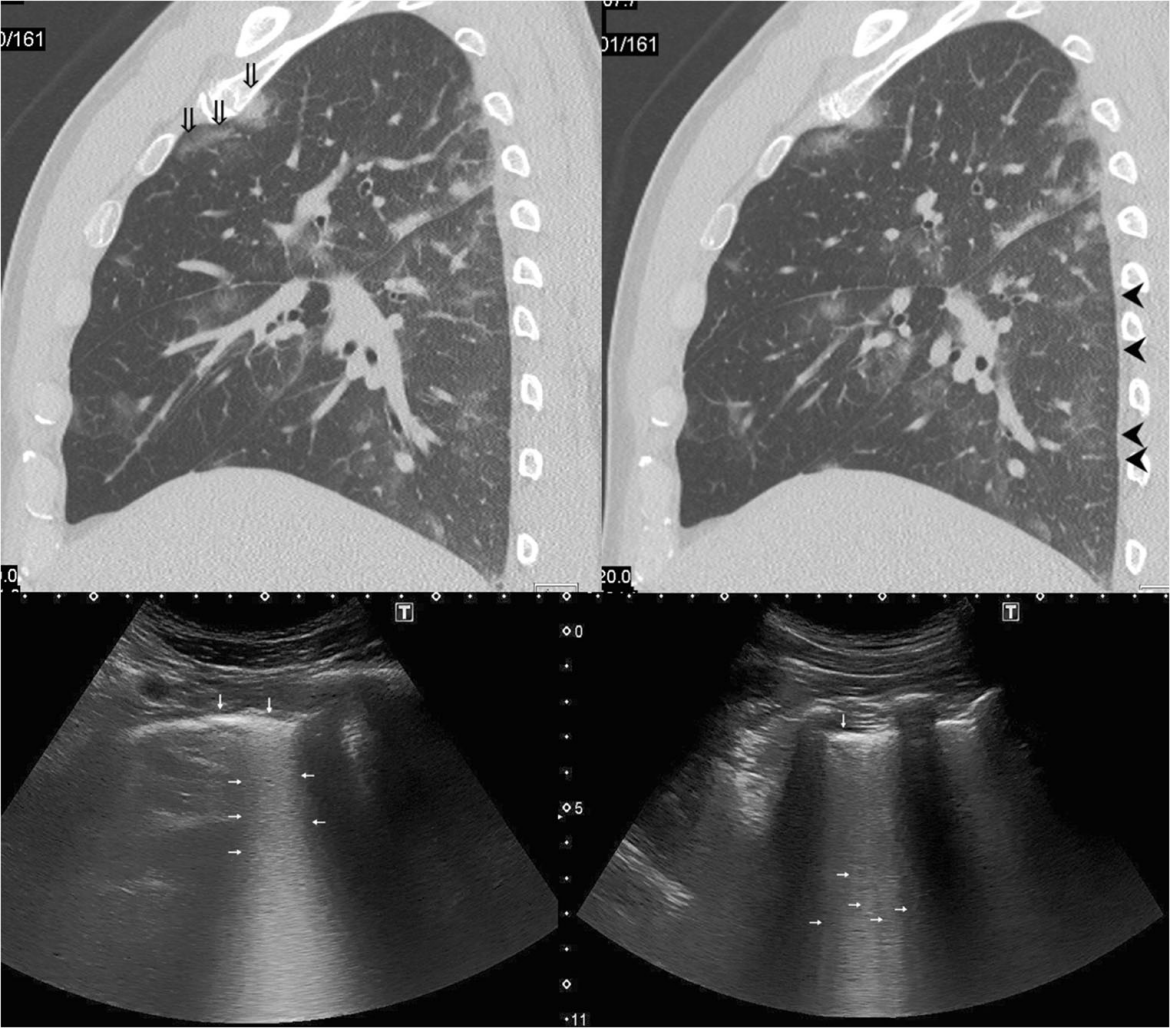
Comparison of exemplary lesions on CT and LUS. Left: GGO in the upper lobe of the right lung (double arrows) yield very densely converging B-Lines (horizontal arrows) that seem to merge into one broad, echogenic vertical artifact arising from the irregular pleural line (vertical arrows). Right: Thickened interlobular septa (arrowheads) are visible on CT; they correlate to B-Lines in LUS (horizontal arrows) that are still distinguishable from one another. The density of B-Lines seems to correlate to the extent of thickening in interlobular septa [7]. All images were obtained on day 17 after symptom onset

**Fig. 3:**
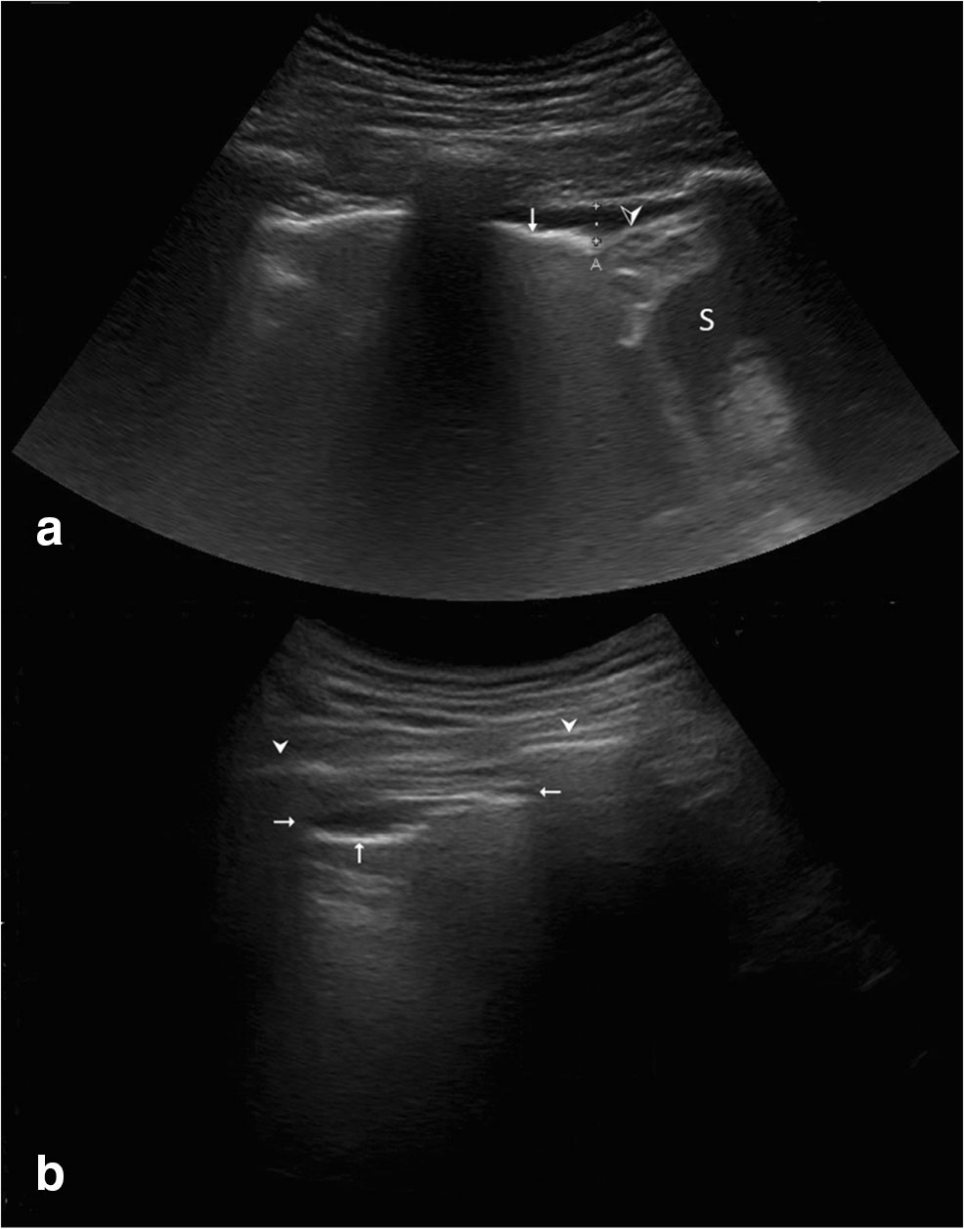
Pleural pathologies observed. Posterior lung scans in the sitting patient. (a) Pleural effusion in the costodiaphragmatic recess in the upright patient, posterior longitudinal view. Distance “A” (dotted line) displays the largest sagittal extent (between chest wall and diaphragm) of this small effusion, which measures 6 mm. Vertical arrow: Visceral pleural line. Arrowhead: Diaphragm. S: Spleen. Note that this effusion was too small to be seen on CT in the supine patient; as fluid collects in the costodiaphragmatic recess in the sitting patient, it was detected using LUS. (b) Pleural plaque in posterior lung scan. Two anechoic lesions (horizontal arrows) are seen adjacent to the parietal pleura. The differential diagnosis of consolidation is dismissed with dynamic visualization of the visceral pleura (vertical arrow) sliding past the stationary lesion; the differential diagnosis of an effusion is dismissed due to the stationary nature of the lesion, (1) not descending into the costodiaphragmatic recess upon inspiration and (2) lack of respiration-dependent expansion and contraction

In 9 patients, we found 3 or more B-Lines (see Figs. 1 and 2) in at least two ICS, in all these cases B-Lines were present bilaterally. Two of these had no other visible pathology other than B-Lines; two had small pleural effusion in their costodiaphragmatic recess up to 6 mm in sagittal extent (see Fig. 3), and five had detectable subpleural consolidation, 4 of which extended longitudinally for more than one ICS. We saw the most severe findings in a patient with severe ARDS (H orowitz Index 80.7mmHg) who showed extensive *tissue-like* and *shred signs* posterolaterally with numerous B-Lines anteriorly.

One other patient had bilateral pleural plaques but no history of exposure to asbestos, silica or other dusts and no signs of interstitial involvement (see Fig. 3b).

In patients with interstitial pathologies, unaffected areas showing A-Lines with up to two b-Lines are more common in anterior regions. Views with 3 or more B-Lines and *tissue* or *shred signs*, if present, were more common in lateral and posterior regions.

### Results of CXR in Comparison to LUS and CT

In 8 out of the 13 patients who had CXR performed, no pathological finding could be detected. In those 8 patients with inconspicuous CXR, 3 also had a CT performed. Only in one of those did the CT scan confirm absence of pulmonary pathology, the other two CTs showed subtle ground glass opacities (GGO) within the lungs. In 5 patients with inconspicuous CXR who did not have CT performed, LUS revealed subtle pulmonary pathologies in 3 patients. One of these was a case of bilateral pleural plaques seen on ultrasound (Fig. 3) but not on CXR. In all cases where CXR did show bilateral opacities, those were also seen on LUS. Conversely, not all pathologies that were detected using LUS were seen on CXR.

### Results of LUS in Comparison to CT

In 8 patients who had received a CT scan of the chest, one did not show any pathologies. Of the other 7 patients, GGO and consolidations were seen in 4 cases in CT, and GGO only without consolidation in another 3 cases. None of the performed CT scans showed signs of PE.

In one case, LUS revealed no interstitial pathology even though GGO were seen in the CT scan and the patient experienced respiratory distress. In one other case, LUS failed to detect interstitial pathology that was seen on CT but revealed a subtle pleural effusion. In the other 5 cases where CT revealed abnormalities, LUS showed corresponding abnormalities (B–Lines, consolidation) in affected intercostal spaces where CT indicated these pathologies. In patients who had a high degree of interstitial pathology indicated by B-lines, consolidations also tended to be more extensive. We would like to point out that 4 patients had very small consolidations that were not revealed on CXR. The patients were asymptomatic, their LUS only revealed A-Lines apart from small *shred signs* and we cannot rule out that we detected preexisting peripheral abnormalities that are not associated with COVID-19.

LUS showed abnormalities corresponding to chest CT in 5 out of 7 cases. LUS showed *any* kind of abnormality in 6 out of 7 cases where CT was abnormal.

## Discussion

This limited retrospective analysis demonstrates our experience with LUS in COVID-19 patients. This descriptive case series should indicate the potential of point-of-care LUS compared to radiographic imaging, particularly compared to CXR under IPC precautions.

We detected three or more B-Lines in at least two ICS in 9 symptomatic COVID-19 patients and never in asymptomatic patients who tested positive for SARS-CoV-2. One patient with respiratory distress who showed interstitial involvement on CT did not have three or more B-Lines per ICS on LUS. As interstitial involvement progresses to ground glass opacities on CT, B-Lines become more numerous and confluent [7] (Fig. 2), the pleural line from which they arise becoming more irregular. Similar results have been described in a recent letter [13], in which the authors detected B-Lines in COVID-19 patients corresponding to GGO on chest CT. Compared to CT, LUS was reliable in detecting signs of interstitial disease in our patients. Scientific consensus suggests that LUS is superior to CXR in detecting interstitial abnormalities [4] which we can neither confirm nor dismiss in COVID-19 due to limited sample size.

LUS in COVID-19 reportedly [13] reveals both small and large consolidations, which we confirm in our series: In 9 patients, we detected consolidations; in 5 cases, they did not extend over more than one ICS. We saw consolidations more frequently in lateral and posterior views, possibly corresponding to less aerated areas. As with any imaging modality, we see the necessity to evaluate imaging results in the context of clinical appearance, since very small consolidations could be detected in otherwise asymptomatic patients.

As reported before [13], pleural effusion is rare in COVID-19. We only detected pleural effusion in three patients. The instances of pleural effusion that we saw were always subtle, ranging no more than a few millimeters in sagittal orientation. Larger effusions in COVID-19 patients might be indicative of bacterial superinfection or cardiac pathologies; however, we did not see any such cases yet. One patient showed pleural plaques on LUS without history of occupational exposure to dusts or preexisting pleural disease. This is, to our knowledge, the first report of pleural plaques associated with COVID-19. However, we cannot conclude whether or not viral disease indeed caused these plaques.

Another pulmonary pathology frequently seen in COVID-19 is pulmonary embolism (PE) [19]. Although none of the patients in our cohort showed PE on contrast-enhanced chest CT, LUS might turn out as a useful diagnostic tool in detecting this condition. On LUS, PE presents as peripheral *tissue-like sign* with absent perfusion (as demonstrated on color or power Doppler) [4, 17]. LUS is a viable alternative in diagnosing PE if CT is contraindicated or unavailable [4, 17], offering similar diagnostic accuracy (see Table 2). Performing CTs in patients under high isolation precautions is difficult and may ultimately delay diagnosis, whereas LUS accelerates pulmonary diagnostics, particularly in acutely ill patients, as compared to radiologic studies [5]. Ultrasound is also useful in detecting concomitant deep vein thrombosis as surrogate indicator for PE in dyspnoeic patients with otherwise normal LUS [8]. The usefulness of ultrasound in COVID-associated PE has however not yet been investigated in large-scale, blinded studies and might be a promising topic for future research. Contrast-enhanced ultrasound (CEUS) even discerns pneumonic consolidations and PE [20, 21] which may be a useful application particularly in COVID-19 where both PE and pneumonic consolidations are frequent findings.

**Table 2:** Overview of systematic reviews and studies of different imaging modalities in COVID and PE. Note that large-scale reviews on LUS in COVID are missing data on sensitivity or specificity, as they mostly rely on case series not reporting this information. Future trials to assess these specifications are desirable. There are also no studies available on LUS in COVID-associated PE. (*CTPA* computed tomography pulmonary angiography, *PIOPED* Prospective Investigation of Pulmonary Embolism Diagnosis criteria, *PISA-PED* Prospective Investigative Study of Pulmonary Embolism Diagnosis criteria)

Study	Modalities	Article type	Pulmonary disease	No. of patients	Findings	Sensitivity [%] (95%-CI)	Specificity [%] (95%-CI)
Kim et al. [14]	CT, PCR	Metaanalysis (63 articles)	COVID	6218	GGO, consolidations	CT: 94 (91–96) PCR: 89 (81–94)	CT: 37 (26–50) PCR: *N.A.*
Smith et al. [15]	LUS	Review (11 articles)	COVID	*N.A.*	B-Lines, consolidations, pleural abnormalities	*N.A.*	*N.A.*
Mohamed et al. [16]	LUS	Review (6 articles)	COVID	122	B-Lines, consolidations, pleural abnormalities	*N.A.*	*N.A.*
Squizzato et al. [17]	LUS, CTPA	Metaanalysis (10 articles)	PE (non-COVID)	887	LUS: subpleural lesions, pleural effusion CTPA: interruption in arterial contrast enhancement	LUS: 87 (79.5–92) CTPA: *N.A.*	LUS: 81.8 (71–89.3) CTPA: *N.A.*
He et al. [18]	CTPA, PIOPED, PISA-PED	Multicenter study	PE (non-COVID)	544	Interruption in arterial contrast enhancement	CTPA: 81.7 PIOPED: 85.1 PISA-PED: 86	CTPA: 93.4 PIOPED: 82.5 PISA-PED: 81.2

In non-COVID patients, lung ultrasound could also discern the severity of acute respiratory distress syndrome (ARDS) [22], to which COVID can progress. We only saw one patient with ARDS who had the most extensive findings regarding the consolidations detected. Large studies correlating lung ultrasound findings to the disease severity of COVID-associated ARDS might be desirable in order to establish this method in this setting.

Conclusively, asymptomatic patients rarely show interstitial involvement (more than three B-Lines per ICS) on lung ultrasound, and the minute consolidations or pleural abnormalities that we did find in asymptomatic patients may or may not be attributable to COVID-19. Patients in respiratory distress show signs of interstitial disease, usually several regions with multiple B-Lines per ICS in asymmetric patterns and varying consolidations.

This brief report is obviously very limited, and as we did not perform all imaging modalities in each patient, we cannot conclude or prove which is superior. We could not systematically analyze LUS due to limited sample size and because we inconsistently performed conventional imaging or invasive diagnostics, based on the patients’ differing disease severity. LUS cannot access the entire lung surface, and one should keep in mind that LUS will not detect abnormalities at all if they do not reach the pleura. Furthermore, ultrasound is generally considered operator-dependent and requires a learning period. We do however see indications that LUS suitable for detecting the subtle interstitial and alveolar pathologies of viral SARS-CoV-2 pneumonia. LUS might be considered especially for use under IPC measures necessary for COVID-19. As cases are expected to rise, we anticipate more extensive use of LUS in COVID-19 as it can be done at bedside under quarantine precautions, accelerates diagnostic imaging [5], and can be repeated numerous times without exposing patients to radiation. We would like to encourage future research to compare LUS to CT in large studies as available literature at this point rarely reports on sensitivity or specificity of LUS (see Table 2).
